# Variations in root architecture traits and their association with organ mass fraction of common annual ephemeral species in the desert of northern Xinjiang

**DOI:** 10.1002/ece3.10908

**Published:** 2024-02-07

**Authors:** Taotao Wang, Bangyan Liu, Xuan Zhang, Mao Wang, Dunyan Tan

**Affiliations:** ^1^ Xinjiang Key Laboratory for Ecological Adaptation and Evolution of Extreme Environment Biology, College of Life Sciences Xinjiang Agricultural University Urumqi China

**Keywords:** interspecific variation, intraspecific variation, leaf mass fraction, root mass fraction

## Abstract

The variation of plant traits is closely related to the trade‐offs between resource acquisition and conservation, as well as the accumulation of biomass. However, there has been a lack of comprehensive insights into the variation patterns, phylogenetic conservatism, and covariation with biomass allocation of root system architecture in desert areas. We examined the root systems of 47 annual ephemeral species and evaluated their biomass allocation and six key root system architecture traits. Our results indicated that the variation in root traits mainly originated from interspecific variation (48.78%–99.76%), but intraspecific variation should not be ignored as to why the contribution rate of root tissue density (RTD) reached 51.22%. The six root traits were mainly loaded on the first and second axes of the principal component analysis (PCA), these traits mainly vary along two dimensions. The highest interspecific variation is in RTD (51.63%) and the lowest in topological index (TI; 5.92%). The intraspecific variation value and range of specific root length (SRL), specific root area (SRA), and RTD were significantly higher than TI (*p* < .05), and they are not limited by phylogenetic relationships (0< *K* < 1, *p* > .05). The SRA is positively correlated with SRL (*r* = .72, *p* < .001) and negatively correlated with RTD (*r* = −.57, *p* < .05). The LMF is positively correlated with SRL, and SRA demonstrated the coordination between water consumption and acquisition. The positive correlation between RMF and MRD indicated the coordination of root carbon investment with exploring soil vertical space. The multi‐dimensional variation of root traits, divergence of RTDs, and convergence of TI are important ecological strategies for annual short‐lived plants to adapt to heterogeneous desert habitats. Meanwhile, these plants achieve optimal access to scarce resources through the high plasticity of resource acquisition (e.g., SRL and SRA) and conservation traits (e.g., RTD), as well as the trade‐offs between them and organ mass fraction.

## Graphical abstract

In the present study, biomass allocation and phylogenetic relationship affect the root system architecture and its variation patterns were investigated in annual ephemerals. Although we found that biomass allocation indicators and trait variation can reflect the adaptive strategies of annual ephemerals, the impact of phylogenetic relationships is equally important.

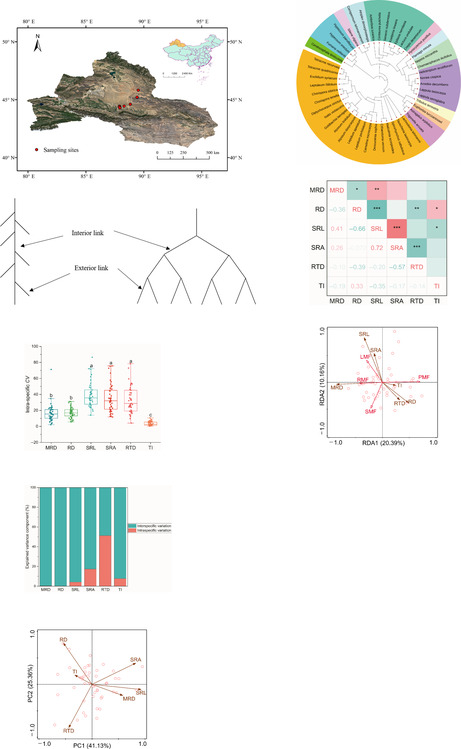

## 1 INTRODUCTION

Root system architecture refers to the morphological traits and branching patterns of the root system in the soil matrix, which play a prominent role in exploring soil space and acquiring resources (Laboski et al., 1998; Lynch, 1995; Tracy et al., 2015). The root morphological traits are closely related to their efficiency in obtaining water and nutrients from the soil and their ability to resist environmental stress (Freschet et al., 2017; Markesteijn & Poorter, 2009; Weemstra et al., 2021). Branching patterns are often described by topological index (TI), and different branching patterns generally represent the internal competition patterns of root system and their adaptability to different soil habitats (Oppelt et al., 2001; Spanos et al., 2008). As a consequence, root system architecture has a profound impact on whole‐plant growth and development, which is the basis for them to adapt to constantly changing environmental conditions (Alvarez‐Flores et al., 2014; Hogan et al., 2020).

The variation of root system architecture traits reflects how they adapt to changing environments (Addo‐Danso et al., 2020; Hogan et al., 2020; Yu et al., 2022). Different species seek to optimize resource acquisition through multiple root system architecture, and this complementary ecological strategy formed through differentiation of resource acquisition strategies within soil profiles may promote species coexistence (Guo et al., 2008; Hogan et al., 2020). The interspecific differences in this plant trait of coexisting species typically increase due to interspecific competition and decrease due to environmental filtering (Weiher et al., 2011). In addition, based on the study of interspecific variation of root traits, some published research have proposed that root traits undergo covariation along a one‐dimensional root economics spectrum (RES), representing a general trade‐off between resource acquisition and resource conservation traits (Prieto et al., 2015; Reich, 2014; Zhou et al., 2018).

However, other research claimed that root system has to cope with more complex environments and undertake diverse ecological functions, so root traits should vary along multiple dimensions (Carmona et al., 2021; Erktan et al., 2018; Kramer‐Walter et al., 2016; Weemstra et al., 2016). These studies have drawn inconsistent conclusions based on the interspecific variation patterns of fine root traits in woody plants (Kong et al., 2019; Weemstra et al., 2016; Yu et al., 2022). Moreover, research on herbaceous plants also focuses more on the inter‐species variation patterns of root traits, with less research on intraspecific variation (Grime, 2006; Yu et al., 2022; Zhou et al., 2018). However, increasing empirically published evidence demonstrated that intraspecific variation is an ecological indicator that cannot be ignored because of the representation of plant response to environmental changes and phenotypic plasticity (Albert, Thuiller, Yoccoz, Douzet, et al., 2010; Defrenne et al., 2019; Siefert et al., 2015). Therefore, it is necessary to further verify the interspecific and intraspecific variation patterns of root traits in herbs, which helps us to have a more comprehensive insight into underground ecological processes.

Species evolution is an important driving factor for root trait variation (Hogan et al., 2020), and this impact may be stronger than environmental factors including climate change and mycorrhizal status, although they have been considered important factors affecting root system architecture variation (Lozano et al., 2020; Maherali, 2017; Valverde‐Barrantes et al., 2017). The root trait phylogenetic conservatism (RTPC) hypothesis suggests that differences between root traits in related species may be smaller compared to phylogenetic structures with weak leaf traits, thereby exhibiting strong phylogenetic conservatism (Liu et al., 2019; Valverde‐Barrantes et al., 2014). Research on morphological traits of fine root on a global scale suggested that specific root length (SRL), root diameter (RD), and other root system architecture traits of woody plants are limited by species evolutionary history so that these pieces of evidence demonstrate similarity in root traits among related species (Kong et al., 2014; Ma et al., 2018; Valverde‐Barrantes et al., 2017; Zhou et al., 2018). However, the diversity of root system functions and the complexity of soil environment may lead to the impact of species evolutionary history on root system architecture traits that is not consistent with the expectations of the RTPC hypothesis (Kramer‐Walter et al., 2016; Wang, Cheng, et al., 2018; Wang, Wang, et al., 2018). Consequently, it is necessary to conduct more empirical research to verify whether phylogenetic relationships have a significant impact on the formation and development of root system architecture.

The root trait variation driven by evolutionary and ecological pressure allows plants to adopt optimal resource acquisition strategies by adjusting resource allocation and trait covariation (Bouma et al., 2001; Freschet et al., 2018; Poorter et al., 2012; Zhou et al., 2019). Specifically, root system may be subjected to environmental stresses such as mechanical damage, soil protozoa grazing, and drought stress during the growth process (Markesteijn & Poorter, 2009; Weemstra et al., 2016). The root system adapts to these environmental conditions with an optimal strategy by coordination or trade‐offs between different root system architecture traits (Lozano et al., 2020; Reich, 2014). In terms of whole plant, those adaptive changes in root system architecture determine the foraging characteristics and how underground resources are acquired and conserved (Alvarez‐Flores et al., 2014; Guo et al., 2008; Hogan et al., 2020), which directly affect the material accumulation and morphogenesis of the aboveground parts of the plant (Dannowski & Block, 2005). Conversely, the development and expansion of roots in soil depend on the carbon fixed by photosynthesis in plant leaves (Willaume & Pagès, 2011). Therefore, plant functional traits are potential covariates that explain biomass allocation, and there may be also coordination or trade‐offs between them (Yin et al., 2019).

Annual ephemeral plants are an important component of desert early spring vegetation in northern Xinjiang, China (Mao & Zhang, 1994). They are a unique plant group with a distinctive life history, which utilizes winter snow melt water and relatively sufficient precipitation in spring to quickly germinate and grow in early spring (Zhang et al., 2020). As a consequence, they can quickly complete their life cycle before the onset of a dry and hot summer climate (Mao & Zhang, 1994; Wang et al., 2021). Through long‐term adaptive evolution, this plant group has formed an ecological strategy suitable for harsh desert environments (Lan & Zhang, 2008; Shi et al., 2006). The most published research have focused on the adaptive characteristics of the aboveground parts of annual ephemerals (Cheng & Tan, 2009; Lu et al., 2015; Mamut et al., 2018; Xiao et al., 2014), with relatively few studies on root systems. In addition, published empirical experiments mainly focus on the impact of environmental factors on the growth and biomass allocation patterns of annual ephemerals (Cheng et al., 2006; Mamut et al., 2019; Qiu et al., 2007; Zhang et al., 2020), with little attention paid to the ecological adaptation of root system architecture of annual ephemerals to the desert environment in the genetic context.

Therefore, this study attempts to solve the following scientific problems by studying the root system architecture traits of 47 annual ephemerals: (i) What are the variation patterns of root architecture traits in annual ephemeral species? (ii) Are they influenced by the phylogenetic relationship of the species? (iii) How do annual ephemerals adapt to desert environments through coordination or trade‐offs between root system architecture traits and biomass allocation?

## 2 MATERIALS AND METHODS

### 2.1 Geography of the study area

The study site is located in the desert (34°09′–49°08′ N and 73°25′–96°24′ E) of the Junggar Basin in northern Xinjiang Uygur Autonomous Region (hereafter Xinjiang), China. It is characterized by drought and little rain in desert habitats, strong environmental heterogeneity, and high seasonal precipitation. The annual average precipitation does not exceed 200 mm, and the annual average temperature is −4 to 9°C, which is a typical continental arid desert climate. Natural vegetation mainly consists of shrubs, perennial herbs, and ephemerals (Ma et al., 2021). Precipitation is mainly concentrated in spring and early summer, and there is relatively stable snow cover in winter, which provides favorable environmental conditions for the successful completion of the life cycle of ephemerals in the region (Shi et al., 2006; Zhang et al., 2020). During the peak growing season of ephemeral plants, their vegetation coverage can reach 40%.

### 2.2 Field investigation and sample collection

**FIGURE 1:**
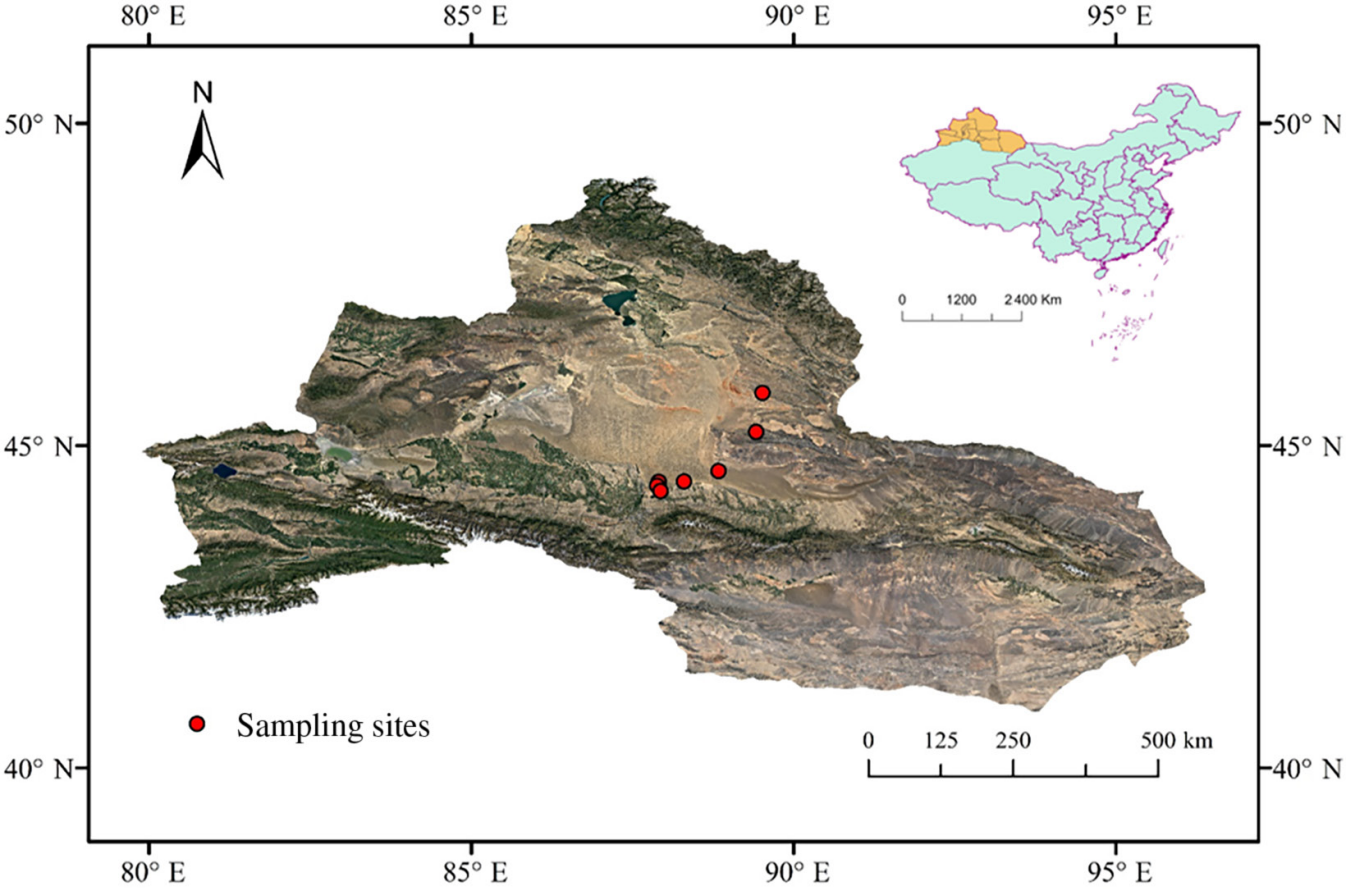
Distribution of sampling sites of annual ephemerals in the desert of northern Xinjiang, China.

The ephemeral plant species are an important component of the sampling area, and their proportion in the community can reach over 50% (Yuan & Tang, 2010). There are a total of 125 species of annual ephemeral plants in northern Xinjiang, accounting for 60% of all ephemeral plant species (Mao & Zhang, 1994). In the 7 sites we investigated (Figure 1), a total of 47 species of annual ephemerals were investigated, accounting for 37.6% of the annual ephemerals in the region, and we selected 10 individuals for each plant species.

Complete plant individuals with vigorous growth were collected from April to May 2022. To avoid the impact of developmental stages, we uniformly sampled at the peak flowering period of each species, and each species is only collected once. However, due to the limitations of species distribution, the number of species collected at each sampling point varies, but usually, at least 10 individuals of one species were collected (Table S1). When the same species appears at different sampling sites, we only collect at one of them. That is to say, when a species repeatedly appears in our set sample, we will not collect it. To eliminate the influence of terrain factors, we have ensured the similarity of the terrain of the seven sampling sites when setting up the quadrat. At each sampling site, a 10 × 10 m quadrat was set, and the plant height and root collar diameter of all individuals of each plant species in the quadrat were investigated, but only species that were flowering at that time, the other species were omitted and they were evaluated next time. To minimize the impact of plant individual size on intraspecific variation (Zheng et al., 2022) and biomass allocation (Poorter et al., 2012), the average value was taken as the standard sample for sampling.

In the quadrat, we selected 10 individuals whose plant height and root collar diameter were similar to the standard samples. The big shovel, small shovel, 30 cm steel ruler, brush, and other tools were used to dig all roots in situ with the “trench method” (Shan et al., 2012). That is, a volume of 10 × 5 × 40 cm trench at a distance of 3–4 cm from the individual plant was dug with a big shovel. Then, the soil around the root was cleared into the trench with a small shovel. When the larger diameter taproot was exposed, to avoid root system damage, we used a steel ruler to continue to clean the soil around the finer root branches. After the root system of the plant was completely exposed, the maximum root depth (MRD) of them was measured in situ with a tape measure and recorded it. After that, the plant individuals were carefully placed in a plastic bag and then transported to store in an ice box back to the laboratory.

### 2.3 Trait measurements and calculations

After washing the impurities on the surface of the collected root systems with distilled water, the relevant parameters were measured. The roots of each plant were scanned using the Epson Perfection V850 Pro Scanner (Epson, Los Alamitos, CA USA), then the scanned root images were numbered and stored in a computer. Each image was analyzed using the Win‐RHIZO Pro 2013 (Regent Instruments Inc., Canada) to measure total root length (TRL), root surface area (RSA), root diameter (RD), and root volume (RV). After scanning, the root system and the aboveground part were put into different empty envelopes respectively and dried to constant weight in an 80°C oven. The dry weight of each part was weighed with the electronic balance (0.1 mg). The specific root length (SRL) was calculated as the ratio of total root length to biomass, the specific root area (SRA) was root surface area to biomass, and the root tissue density (RTD) was biomass to root volume. The root mass fraction (RMF) was the proportion of root biomass to whole‐plant biomass, the leaf mass fraction (LMF) was leaf biomass to whole‐plant biomass, the stem mass fraction (SMF) was stem biomass to whole‐plant biomass, and the reproduction mass fraction (PMF) was reproduction biomass to whole‐plant biomass (Table 1).

**TABLE 1:** Root system architecture traits and plant organ mass fraction measured in this study.

Trait	Abbreviation	Meaning	Unit
Maximum root depth	MRD	Root system can reach the maximum depth of the soil	cm
Root diameter	RD	The average diameter of the root system	mm
Specific root length	SRL	Root biomass per unit of root length	cm·g^−1^
Specific root area	SRA	Root biomass per unit root surface area	cm^2^·g^−1^
Root tissue density	RTD	Root biomass per unit root volume	g·cm^−3^
Topological index	TI	The branching pattern of the root system	
Root mass fraction	RMF	The proportion of root biomass to total plant biomass	g·g^−1^
Leaf mass fraction	LMF	The proportion of leaf mass to total plant biomass	g·g^−1^
Stem mass fraction	SMF	The proportion of stem mass to total plant biomass	g·g^−1^
Reproduction mass fraction	PMF	The proportion of reproductive organ mass to total plant biomass	g·g^−1^

### 2.4 Calculation of root topology

**FIGURE 2:**
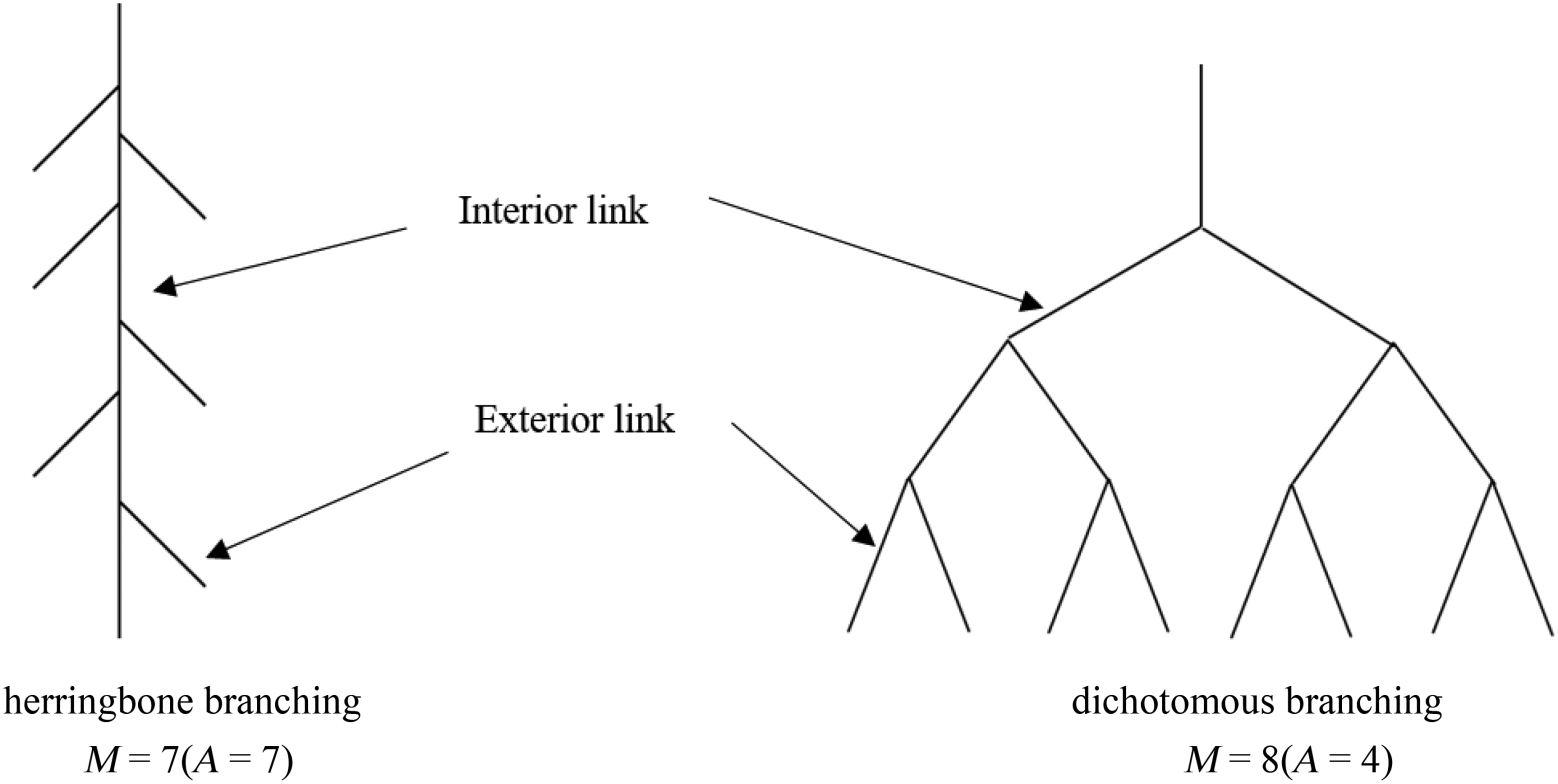
The schematic diagram of topological structure of root system. Altitude (*A*) is the number of connections from the root collar to one of the longest channels at the root tip. Magnitude (*M*) is the total number of all exterior connections.

According to the root branching theory proposed by Fitter and Stickland (1991), there are two extreme types of root branching patterns: (i) herringbone branching with relatively simple branches and (ii) dichotomous branching with relatively complex branches. This theory applies river branching theory to the study of root branching patterns, using topological index (TI) to describe the branching patterns of different root systems. The TI was calculated as the ratio of lgA to lgM (Figure 2). Altitude (*A*) is the number of connections from the root collar to one of the longest channels at the root tip. Magnitude (*M*) is the total number of all exterior connections. If TI is 1, the root system is a herringbone branching, and if TI is 0.5, it is a dichotomous branching.

### 2.5 Constructing the phylogenetic tree

We have collected 47 species of annual ephemerals from 13 families, 41 genera, and deserts in northern Xinjiang, China. The *Flora of China* section of the *iplant* website (http://www.iplant.cn/) is used to retrieve the latest revised Latin scientific names of various species based on their Chinese scientific names. The plantlist package and V. PhyloMaker package were used to construct an evolutionary tree based on the APG IV system in R 4.2.1 (Jin & Qian, 2019; Zhou et al., 2018). Visualization of phylogenetic tree was constructed with the help of online software iTOL (https://itol.embl.de/tree/). Before detecting phylogenetic signals, the *multi2di()* function in the ape package was used to convert the evolutionary tree into a binary tree (Paradis et al., 2004).

### 2.6 Data analysis

This study calculated the average value of root system architecture traits for each species, and the trait values for each species were derived from 10 individuals. We calculated the variation coefficient (CV, Standard error divided by average and multiplied by 100%) of each root system architecture trait. One‐way ANOVA analysis was used to examine the differences in intraspecific variation values of each trait. In addition, the lme4 package in R 4.2.1 was used to determine the optimal mixed linear model, and the *lmer()* function is used to establish the model. The *glmm. hp()* function in the glmm. hp package was used for variance decomposition, and the proportion between variance components represents the proportional contribution of each scale change. Blomberg's *K* value is used to test phylogenetic signals of six root system architecture traits (Blomberg et al., 2003). The *ReorderData()* function in the evobiR package was used to rearrange the root system architecture trait data in the order of the evolutionary tree, and then the *phylosig()* function in the phytools package in R 4.2.1 was used to calculate the Blomberg's *K* value, which was set to 999 times repeatedly (Kraft et al., 2010; Revell, 2012). Spearman's correlation analysis was used to test the relationship between various traits. In addition, principal component analysis (PCA) was conducted on six root system architecture traits. The PC1 and PC2 could usually explain a higher proportion of variance, and these scores could be used as substitutes for RES in all subsequent analyses. To understand the variation patterns between individual traits and the plant resource economics spectrum, the correlation between PC1 and PC2 scores and each trait was calculated again. In this way, the relative contribution of each trait to PC1 and PC2 could be estimated (Li et al., 2022). Redundancy analysis (RDA) was conducted on the relationship between root system architecture traits and the proportion of plant organ biomass in Canoco5.0 software. Before conducting RDA, Detrended Correspondence Analysis (DCA) was first performed based on the plant organ mass fraction. The results of DCA indicated that the gradient length of the sorting axis is 1.1 (<3), and thus RDA analysis was chosen to further analyze the relationship between the root system traits and the plant organ mass fraction. Origin 2021 (Origin Lab Corporation, Northampton, MA, USA) was used to exert data visualization.

## 3 RESULTS

### 3.1 The variation pattern of root system architecture traits

**FIGURE 3:**
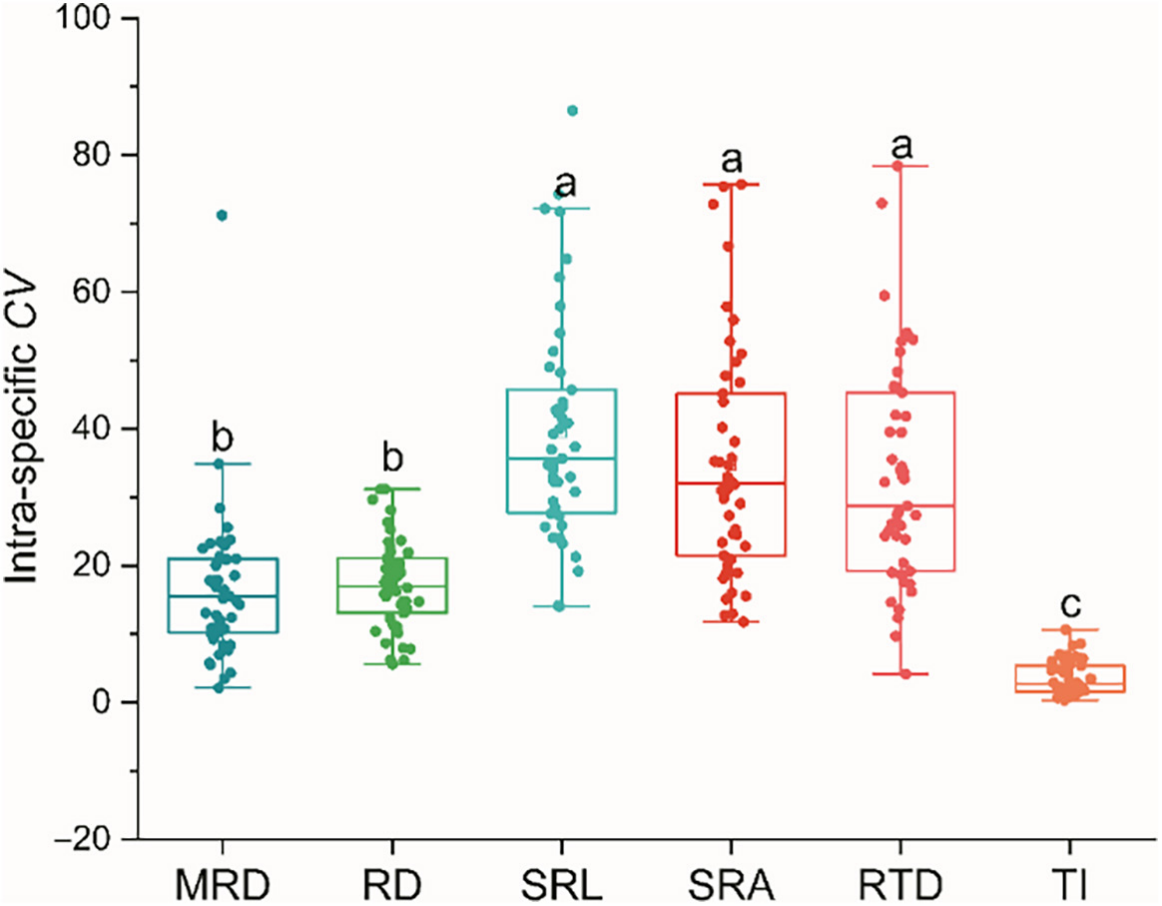
Intraspecific variation coefficients in root system architecture traits of 47 annual ephemerals in the desert of northern Xinjiang, China. Different lowercase letters indicate significant differences (*p* < .05). Each point in the graph represents a species, and each species is calculated from the average of 10 individuals. The meanings of MRD, RD, SRL, SRA, RTD, and TI are shown in Table 1.

**FIGURE 4:**
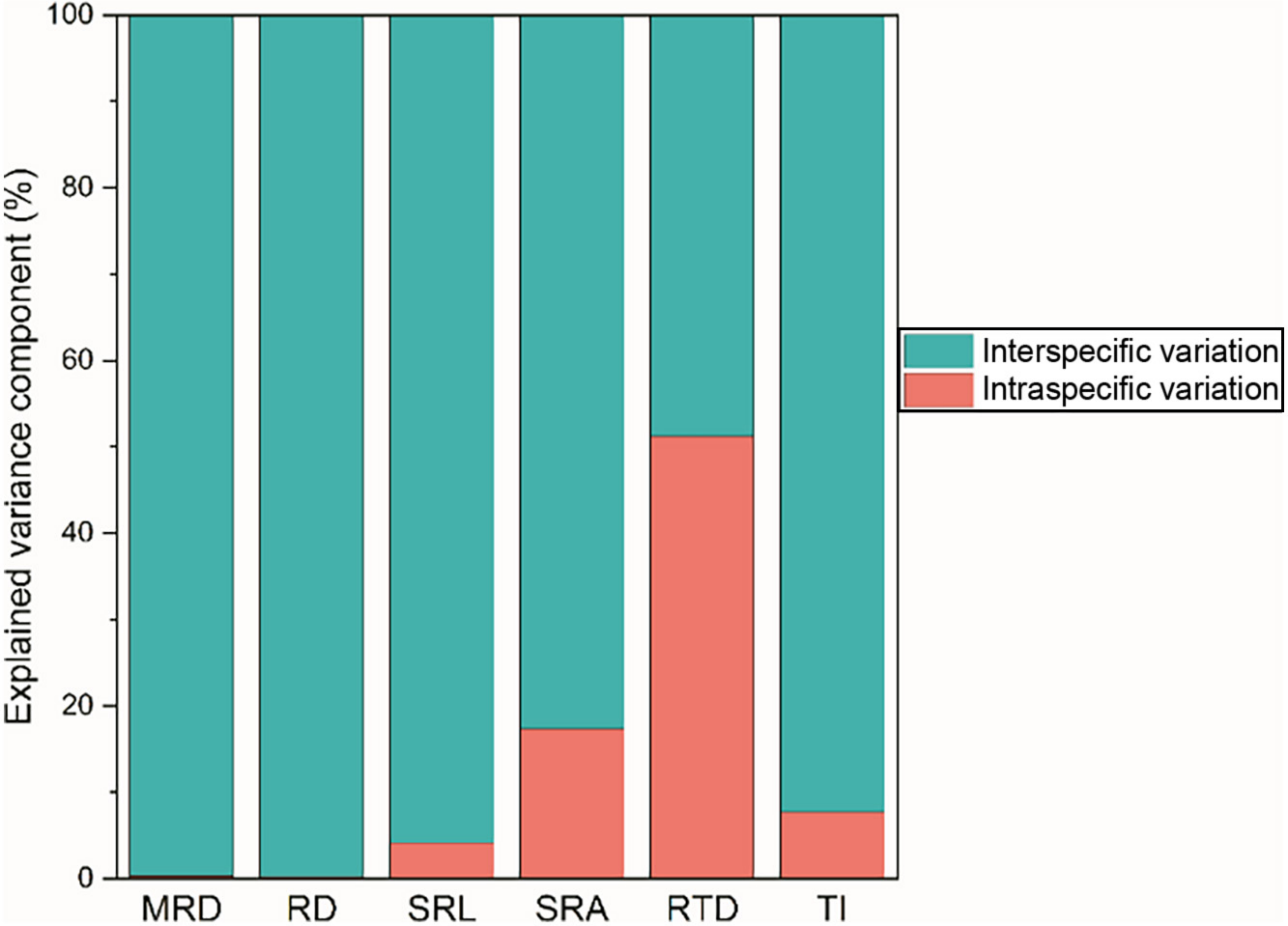
Variance partitioning of different fine root traits at interspecific and intraspecific scales. The meanings of MRD, RD, SRL, SRA, RTD, and TI are shown in Table 1.

Interspecific variation (*CV*_inter_) can reflect the long‐term adaptation of the root system to the environment, while intraspecific variation (*CV*_intra_) is an important indicator of trait plasticity. The *CV*_inter_ of RTD is the highest at 51.63%, while TI is the lowest at 5.92% (Table 2). The *CV*_intra_ ranges of SRA, SRL, and RTD are relatively large, ranging from 11.77% to 75.76%, 14.02% to 86.51%, and 4.17% to 78.45%, respectively. However, the *CV*_intra_ range of TI is the smallest, ranging from 0.30% to 10.62% (Figure 3). Moreover, the intraspecific variation values of the first three are significantly higher than those of the latter (Figure 3; *p* < .05). Interestingly, based on the analysis results of mixed linear models and variance decomposition, we found that the interspecific variation of MRD, RD, SRL, SRA, and TI contributed more to the total variation than the intraspecific variation, while the contribution of RTD was exactly the opposite (Figure 4).

**TABLE 2:** Characteristics and interspecific variation coefficients of root system architecture of 47 annual ephemerals in the desert of northern Xinjiang, China.

Trait	Abbreviation	Mean (±SE)	Max	Min	CV (%)
Maximum root depth (cm)	MRD	14.27 ± 6.06	29.69	3.71	42.46
Root diameter (mm)	RD	0.36 ± 0.12	0.74	0.17	32.45
Specific root length (cm·g^−1^)	SRL	1209.19 ± 600.42	3262.09	294.54	49.65
Specific root area (cm^2^·g^−1^)	SRA	123.17 ± 46.92	286.09	24.44	38.10
Root tissue density (g·cm^−3^)	RTD	1.41 ± 0.73	3.59	0.55	51.63
Topological index	TI	0.95 ± 0.06	1.00	0.77	5.92

### 3.2 Principal component analysis of root system architecture traits

**FIGURE 5:**
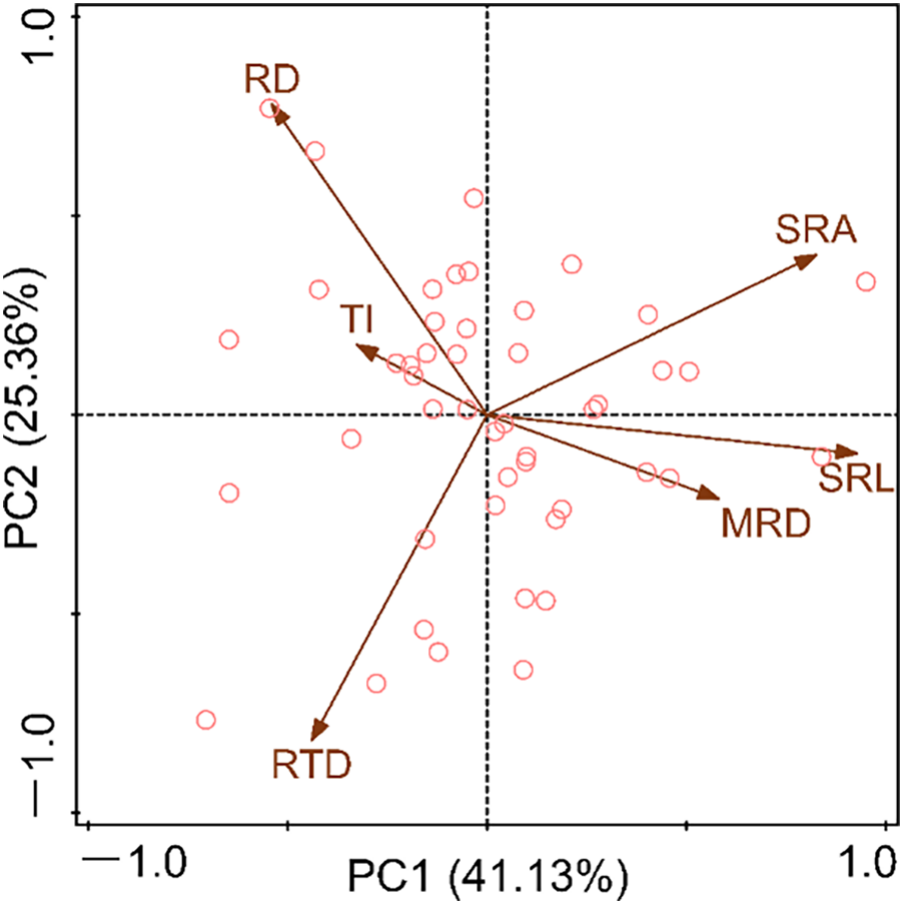
Principal component analysis of root system architecture traits. Each circle in the graph represents a species, and each species is calculated from the average of 10 individuals. The meanings of MRD, RD, SRL, SRA, RTD, TI, RMF, LMF, SMF, and PMF are shown in Table 1.

According to the results of (PCA; Figure 5), the resource utilization strategies of species with different root system architecture can be revealed. The PCA results of root system architecture traits showed that the first two axes together explained 66.49% of the variance, while PC1 and PC2 explained 41.13% and 25.36% of the variance, respectively (Figure 5). The contribution of SRA, SRL, MRD, and TI to PC1 were greater than those to the PC2, while RD and RTD are exactly the opposite (Table 3). The species distributed on the right side of PC1 have higher SRL and MRD, while the species on the left side have higher TI. In addition, species with higher RTD are located below the PC2 axis, while species with higher RD are located above (Figure 5).

**TABLE 3:** Correlation coefficients between individual traits and the scores of the first and second principal components in root economics spectrum.

Axis	Root system architecture traits					
	MRD	RD	SRL	SRA	RTD	TI
PC1	0.56***	−0.50**	0.91***	0.79***	−0.32*	−0.42**
PC2	−0.26	0.78***	−0.23	0.34*	−0.80***	0.25

*Note*: The meanings of MRD, RD, SRL, SRA, RTD, and TI are shown in Table 1.

* *p* < .05;

** *p* < .01;

*** *p* < .001.

### 3.3 Phylogenetic signal of root system architecture traits

**FIGURE 6:**
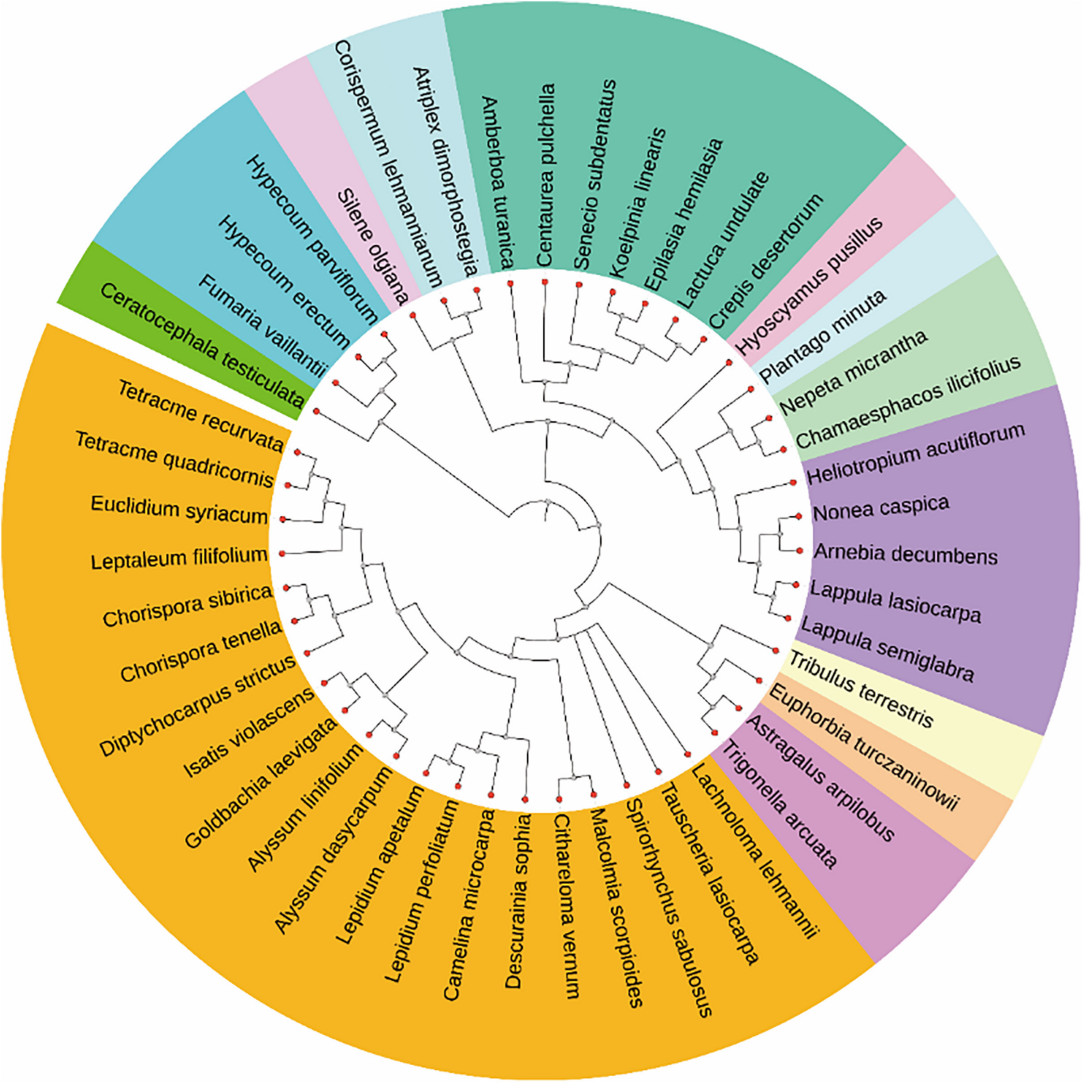
Phylogeny of 47 annual ephemerals based on APG IV in the desert of northern Xinjiang, China. Species from the same family are indicated by the same color.

We constructed a phylogenetic tree of annual ephemerals in this study based on the APG IV (Angiosperm Phylogeny Group) plant classification system (Figure 6). Blomberg's *K* values for six root system architecture traits were immediately detected (Table 4). The results showed that the *K* value of MRD was 0.409, showing a significantly and weakly phylogenetic signal (0 < *K* < 1, *p* < .05). However, phylogenetic signals were not detected for the other five root system architecture traits (0 < *K* < 1; *p* > .05).

**TABLE 4:** Phylogenetic signals of root system architecture traits of 47 annual ephemerals in the desert of northern Xinjiang, China.

Trait	Abbreviation	Blomberg's *K* value	*p*‐value
Maximum root depth (cm)	MRD	0.409	.006
Root diameter (mm)	RD	0.211	.729
Specific root length (cm·g^−1^)	SRL	0.301	.209
specific root area (cm^2^·g^−1^)	SRA	0.206	.808
Root tissue density (g·cm^−3^)	RTD	0.278	.313
Topological index	TI	0.166	.929

### 3.4 Linkages of root system architecture traits with plant organ mass fraction

**FIGURE 7:**
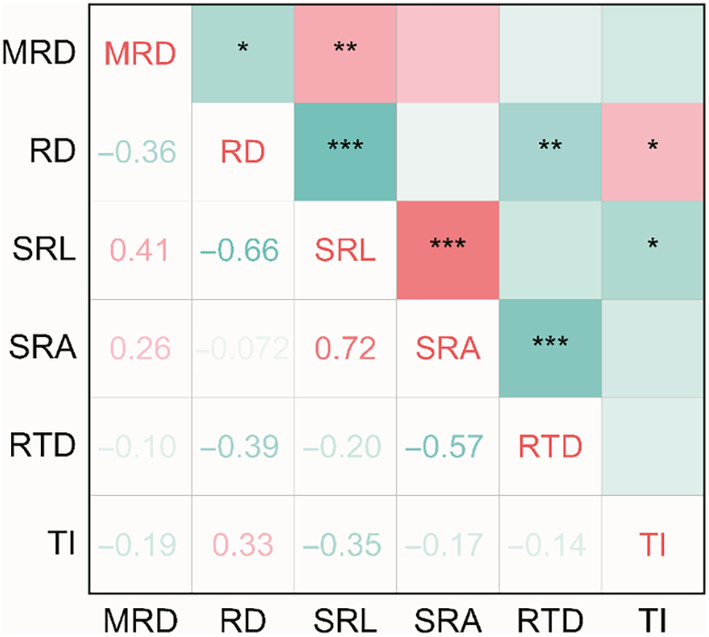
Correlation of root system architecture traits of annual ephemerals in the Northern desert of Xinjiang, China. Green indicates negative correlation and red indicates positive correlation. *p < .05; **p < .01; ***p < .001. The meanings of MRD, RD, SRL, SRA, RTD, TI, RMF, LMF, SMF, and PMF are shown in Table 1.

**FIGURE 8:**
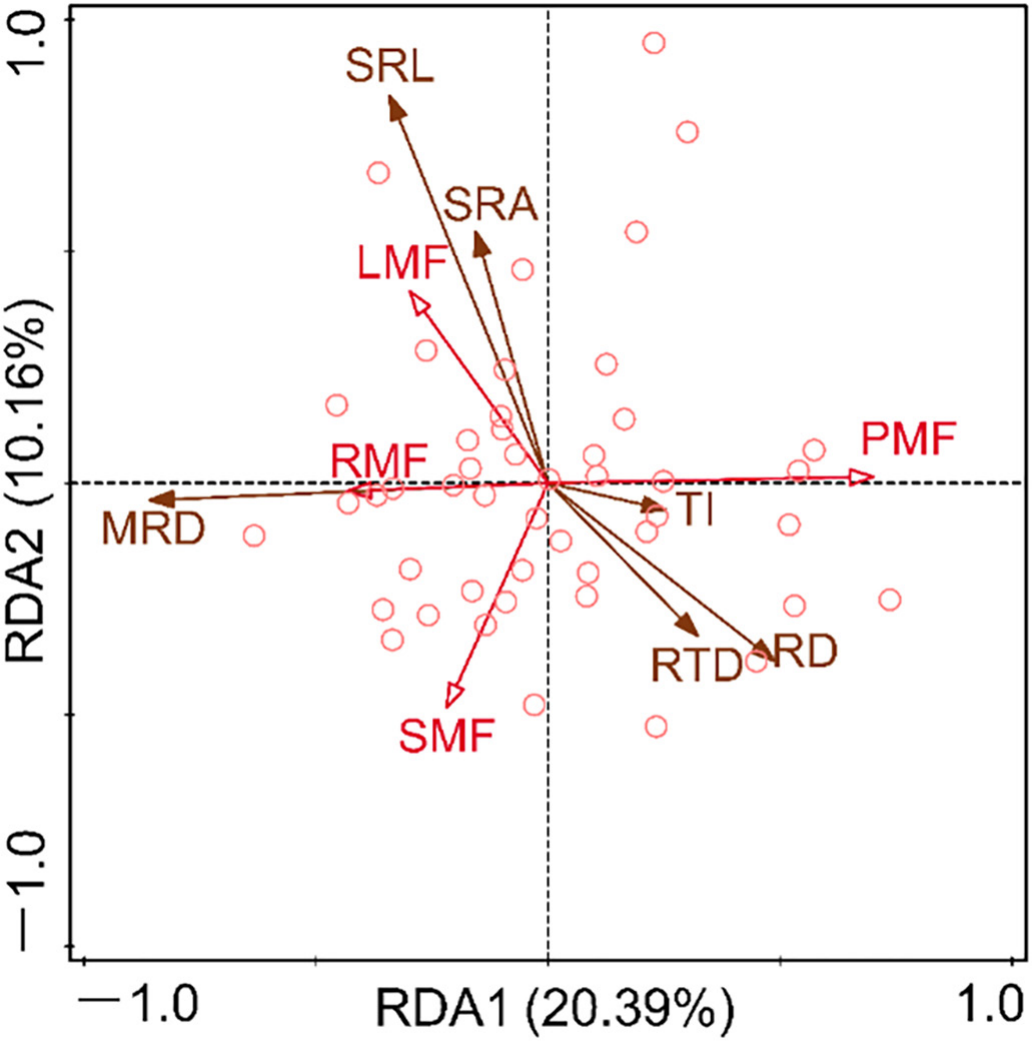
Redundancy analysis (RDA) of root system architecture traits and organic mass fraction. Each circle in the graph represents a species, and each species is calculated from the average of 10 individuals. The meanings of MRD, RD, SRL, SRA, RTD, TI, RMF, LMF, SMF, and PMF are shown in Table 1.

We analyzed the covariation patterns among different root system architecture traits (Figure 7) and relationship of root system architecture traits with organ mass fraction (Figure 8). The results showed a significant positive correlation between MRD and SRL (*r* = .41, *p* < .01), a significant negative correlation with RD (*r* = −.36, *p* < .05). The RD was negatively correlated with SRL and RTD (*r* = −.66, *p* < .001; *r* = −.39, *p* < .05), and positively correlated with TI (*r* = .33, *p* < .05). The SRL was positively correlated with SRA and negatively correlated with TI (*r* = .72, *p* < .001; *r* = −.35, *p* < .05). The negative correlation between SRA and RTD was observed (*r* = −.57, *p* < .05).

Redundancy analysis (RDA) was used for constraint ranking of root system architecture traits and plant organ mass fraction (Figure 8). The results showed that the eigenvalues of the first two axes were 0.2039 and 0.1016, respectively, accounting for 30.55% of the variation in plant organ biomass proportion. The LMF is positively correlated with SRL and SRA and negatively correlated with RD and RTD. The RMF is positively correlated with MRD and negatively correlated with RD and RTD.

## 4 DISCUSSION

### 4.1 The differences in variation patterns of different root traits enhance the adaptability of root system

The interspecific variation of root traits can realistically reflect the response of species to environmental changes and resource competition (Carmona et al., 2021; Erktan et al., 2018; Weemstra et al., 2016). In the present study, the degree of *CV*_inter_ of the six root system architecture traits is distinct (Table 2). Among them, the RTD is the highest, and the TI is the lowest (Table 2). The results manifested that the RTD of annual ephemerals showed a divergent adaptation, while the branching pattern (TI) showed a convergent adaptation due to the combined effects of environmental filtering and similarity constraints (Grime, 2006). The divergence adaptation of RTD is conducive to these plants to reduce biological competition by adopting different root resource defense strategies when dealing with highly heterogeneous desert environments (Lan & Zhang, 2008; Tjoelker et al., 2005; Xu et al., 2021). Meanwhile, the convergence of root branching patterns caused by environmental filtering towards more simple herringbone branching patterns can effectively reduce the carbon investment cost of underground root construction, allowing annual ephemerals to allocate more resources to the growth of the aboveground parts (Qiu et al., 2007; Spanos et al., 2008; Tsakaldimi et al., 2009). In addition, the results of PCA indicated that the interspecific variation of root traits in annual ephemeral species varies along a multidimensional root economic space, rather than a one‐dimensional root economic spectrum (Figure 5; Table 3). Specifically, the species located on the right side of the PC1 axis have higher MRD and SRL, and thus they have efficient resource acquisition strategies. Because they are usually considered to be related to the ability of the root system to explore soil space and water absorption efficiency, respectively (Markesteijn & Poorter, 2009; Pregitzer et al., 2002). Species located on the upper side of the PC2 axis have higher RD, which may indicate their resource acquisition strategies associated with arbuscular mycorrhizal fungi (AMF; Bergmann et al., 2020; Carmona et al., 2021). Because mycorrhizal fungal colonization can assist the root system in nutrient absorption, a thicker diameter often implies higher mycorrhizal colonization (Kong et al., 2014; Valverde‐Barrantes et al., 2017). Moreover, research of Shi et al. (2006) showed that some species located on the upper side exhibited AMF colonization (such as *Koelpinia linearis*, *Atriplex dimorphostegia*, *Hypecoum parviflorum*, and *Astragalus arpilobus*). Finally, species located below the PC2 axis have higher RTD and should have stronger resource conservative capabilities; species with high RTD have been proven to play a crucial role in resisting environmental stress and improving root lifespan (Eissenstat, 1991; Zhou et al., 2018).

Intraspecific variation refers to the differences in genetic material and phenotypic characteristics between different botany individuals from the same species (Albert, Thuiller, Yoccoz, Soudant, et al., 2010). In the present study, although interspecific variation dominates the total variation of root traits, the results of variance decomposition indicated that the contribution rate of intraspecific variation of RTD exceeded that of interspecies, reaching 51.22%. It is worth noting that even if we have minimized the impact of individual size during sampling, the above results still appear. This result further indicated that intraspecific variation is essential in ecological research (Defrenne et al., 2019; Jung et al., 2014). Studying intraspecific variations, especially in functional traits related to roots, can greatly enhance our understanding of the potential of plants to respond to different environmental conditions (Hajek et al., 2013). Specifically, in our investigation, the value and range of *CV*_intra_ in root architecture traits related to the acquisition of resources (e.g., SRL, SRA) and resistance to environmental (e.g., RTD) interference is relatively large, showing high plasticity and divergence in plasticity (Figure 3). This may be because the annual ephemeral species collected in this study are all distributed in heterogeneous desert habitats with low species richness and relatively scarce resources, which causes some species to exhibit high plasticity in root resource acquisition or defense traits (Siefert et al., 2015). Specifically, the high plasticity of these traits is crucial for them to adapt to the desert environment, because when resource availability changes rapidly in time and space, high phenotypic plasticity represents that plants have certain adaptive advantages (Hajek et al., 2013). Additionally, this divergence in the plasticity of resource acquisition traits and defense traits of different species may help plant individuals (even different individuals from the same species) occupy different ecological niches (Bu et al., 2017), thus promoting the coexistence of annual ephemeral plants in limited resource habitats.

The driving factors of ecology and evolution shape the variation of plant traits (Reich, 2014). The present study, a significant weak signal was found for Maximum root depth (MRD; Table 4). This indicated that species with closer genetic relationships adopt more similar strategies when exploring soil vertical space. The phylogenetic conservation of MRD is beneficial for different populations to establish in different ecological niches in the same habitat, as the vertical distribution of water resources in the soil is extremely heterogeneous in desert environments (Kirschner et al., 2021). Our results further strengthen the viewpoint that evolution is one of the main driving forces shaping changes in underground traits (Reich, 2014; Yu et al., 2022). In addition, in the present study, no significant phylogenetic signals were detected for the other root system architecture traits, and SRL, SRA, and RTD were not significantly affected by phylogenetic relationships among species (Kramer‐Walter et al., 2016). These traits break free from the constraints of plant phylogeny and exhibit high plasticity, which is beneficial for desert plants in dealing with highly variable and unpredictable desert environmental conditions (Figure 3).

### 4.2 The coordinated changes between root system architecture traits and biomass allocation optimize adaptive strategy

In the present study, the correlation analysis results between root traits showed that the positive correlation between SRL and SRA was observed (Figure 7), indicating a synergistic change between SRL and SRA, which means that the root length per unit carbon investment is longer and the root surface area is also larger (Wang, Cheng, et al., 2018; Wang, Wang, et al., 2018). The greater the SRL and SRA, the higher the efficiency of roots in exploring soil space and absorbing soil water and nutrients under unit carbon investment (Isaac et al., 2017). Some studies have found a significant negative correlation between SRL and RTD (Lozano et al., 2020; Xu et al., 2021), but in this study, there was no significant correlation between SRL and RTD (Figure 7). This may be due to the complexity of the soil environment and the diversity of root functions (Han & Zhu, 2021), resulting in the expression of root traits typically characterized by acquisition (e.g., SRL) not necessarily being trade‐off with conservative root traits (e.g., RTD; Weemstra et al., 2021). Additionally, there was a significant negative correlation between RD and RTD (Figure 7), which is consistent with studies on root absorption in woody plants (Holdaway et al., 2011; Weemstra et al., 2016; Withington et al., 2006). Moreover, there was a significant negative correlation between SRA and RTD in this study (Figure 7). This indicated that there is a trade‐off between nutrient uptake efficiency and resistance to environmental disturbances in the roots of annual ephemerals.

For the whole plant, plant individuals can achieve a balance between resource acquisition and allocation through coordinated changes in biomass allocation and morphological characteristics (Chapin, 1991; Freschet et al., 2018; Nicotra et al., 2010). Therefore, the RDA was used to examine the relationship between the proportion of plant organ biomass fraction and root system architecture traits. The results showed that LMF is positively correlated with SRL and SRA, while negatively correlated with RD and RTD (Figure 8). This indicated that plants could mitigate water and water loss caused by high LMF by improving the absorption efficiency of root water and nutrient (Isaac et al., 2017; Wang, Cheng, et al., 2018; Wang, Wang, et al., 2018). Specifically, the LMF is closely related to the water consumption capacity of plants, and the higher the LMF, the greater the water consumption and demand of its leaves (Yin et al., 2019). Moreover, the results of this study indicated a negative correlation between LMF and RTD (Figure 8). This may be because RTD is closely related to the resource conservation ability of the root system (Bergmann et al., 2020; Carmona et al., 2021), plants must achieve a balance between resource conservation and consumption among organs through the trade‐off between LMF and RTD. Interestingly, a positive correlation was observed between RMF and MRD (Figure 8), which further indicated that the exploration of soil vertical space by roots depends on the biomass investment of plants in the roots. Furthermore, the negative correlation between RMF and RD was observed (Figure 8). Roots with high root diameters have been proven to be effective resistance to mechanical damage, herbivores, and drought stress (Kong et al., 2014; Weemstra et al., 2016; Withington et al., 2006). According to the functional equilibrium hypothesis, the increase in root biomass is due to resource constraints on root growth (Freschet et al., 2018; Poorter et al., 2012). Therefore, when the scarcity of underground resources leads to an increase in root biomass investment, what the root system most needs is to increase the ability to explore resources or the efficiency of acquiring resources, rather than resisting stress.

## 5 CONCLUSION

The results of this study suggested that the root architecture traits of annual ephemeral species vary along multiple dimensions, and different species adopt advantageous resource acquisition strategies. In addition, compared to soil spatial exploration methods (such as TI), traits related to resource acquisition and preservation (such as SRL, SRA) have higher plasticity, and there is a general coordination or trade‐off between the latter. Among the six root system architecture traits, only the MRD was phylogenetic conserved, species phylogenetic relationships have limitations on their variation. The LMF achieved a balance between water consumption and water acquisition through collaboration with SRL and SRA. The coordination between RMF and MRD may be a resource strategy for root systems to explore soil space based on carbon investment.

## AUTHOR CONTRIBUTIONS

**Taotao Wang:** Data curation (lead); formal analysis (lead); investigation (lead); methodology (equal); writing – original draft (lead); writing – review and editing (supporting). **Bangyan Liu:** Data curation (equal); investigation (equal). **Xuan Zhang:** Data curation (equal); investigation (equal). **Mao Wang:** Funding acquisition (lead); resources (equal); supervision (equal); writing – review and editing (equal). **Dunyan Tan:** Conceptualization (lead); supervision (equal); writing – review and editing (equal).

## CONFLICT OF INTEREST STATEMENT

The authors declared no potential conflicts of interest with respect to the research, authorship, and publication of this article.

## Supporting information


Table S1.

## Data Availability

The data that support the findings in the present study are available at: https://osf.io/4gy6u/?view_only=aaafdf80ca304082ad64522d0d5629ce.
