# Correction to “Variations in root architecture traits and their association with organ mass fraction of common annual ephemeral species in the desert of northern Xinjiang”

**DOI:** 10.1002/ece3.11112

**Published:** 2024-03-13

Wang, T., Liu, B., Zhang, X., Wang, M., & Tan, D. (2024). Variations in root architecture traits and their association with organ mass fraction of common annual ephemeral species in the desert of northern Xinjiang. Ecology and Evolution, 14, e10908.

In paragraph 3 of the “Materials and Methods” section, the text “The glmm.hp() function in the glmm.hp package was used for variance decomposition, and the proportion between variance components represents the proportional contribution of each scale change.” was incorrect. This should have read as follows: “The glmm.hp() function in the glmm.hp package was used for variance decomposition, and the proportion between variance components represents the proportional contribution of each scale change (Lai et al., 2022, 2023).”

We apologize for this error.
