# Supplementary material for: Variations in root architecture traits and their association with organ mass fraction of common annual ephemeral species in the desert of northern Xinjiang
Source: Ecol Evol. 2024 Feb 7;14(2):e10908. doi: 10.1002/ece3.10908 (PMC10847883; doi:10.1002/ece3.10908)
Supplement: Supplementary file 1 — Table S1. [file ECE3-14-e10908-s001.docx]

Table S1 Distribution information of collected species at various points

| Number | Longitude(E) | Latitude(E) | Collected species (47 species in total) |
| --- | --- | --- | --- |
| 1 | 87.9081 | 44.4351 | *Descurainia Sophia*; *Chamaesphacos ilicifolius*  *Hypecoum erectum*; *Malcolmia scorpioides*;  *Centaurea pulchella*; *Atriplex dimorphostegia*;  *Heliotropium acutiflorum*; *Alyssum dasycarpum*;  *Alyssum linifolium* (9 species in total). |
| 2 | 87.8828 | 44.3772 | *Corispermum lehmannianum*; *Crepis desertorum*;  *Tribulus terrestris*; *Nonea capsica*; *Nepeta micrantha*;  *Epilasia hemilasia*; *Leptaleum filifolium*;  *Tetracme quadricornis*; *Euphorbia turczaninowii*;  *Isatis violascens*; *Hypecoum parviflorum*;  *Arnebia decumbens* (12 species in total). |
| 3 | 88.2993 | 44.4416 | *Cithareloma vernum*; *Trigonella arcuate*;  *Lachnoloma lehmannii*; *Silene olgiana*;  *Plantago minuta*; *Lappula lasiocarpa*  (7 species in total). |
| 4 | 89.5176 | 45.8171 | *Chorispora sibirica*; *Lepidium apetalum*;  *Lappula semiglabra* (3 species in total). |
| 5 | 88.8347 | 44.6063 | *Koelpinia linearis*; *Hyoscyamus pusillus*;  *Astragalus arpilobus*; *Spirorhynchus sabulosus*;  *Senecio subdentatus*; *Tetracme recurvata*;  *Lactuca undulate* (7 species in total). |
| 6 | 87.9362 | 44.2931 | *Lepidium perfoliatum*; *Chorispora tenella*;  *Camelina macrocarpa* (3 species in total). |
| 7 | 87.5662 | 43.7985 | *Fumaria vaillantii*; *Goldbachia laevigata*;  *Ceratocephala testiculata*; *Euclidium syriacum*;  *Diptychocarpus strictus*; *Tauscheria lasiocarpa*  (6 species in total). |
